# Hepatic small vessel neoplasm case report: A surveillance conundrum

**DOI:** 10.1016/j.ijscr.2021.105742

**Published:** 2021-03-11

**Authors:** Patricia Mulholland, Ian Y. Goh, Anna Sokolova, Cheng Liu, Mehan Siriwardhane

**Affiliations:** a Department of General Surgery, Mater Hospital, Brisbane, Australia; b Faculty of Medicine, University of Queensland, Brisbane, Australia; c Pathology Queensland, Royal Brisbane and Women's Hospital, Brisbane, Australia; d Mater Pathology, Mater Hospital, Brisbane, Australia; e QIMR Berghofer Medical Research Institute, Brisbane, Australia

**Keywords:** Case report, HSVN, Hepatobiliary, Neoplasia, Hepatic small vessel neoplasm

## Abstract

**Introduction:**

Hepatic small vessel neoplasm (HSVN) is a recently described vascular neoplasm of the liver that can mimic hepatic angiosarcoma (AS) because of its infiltrative nature but is considered benign or, at most, low-grade. HSVN appears to demonstrate a benign clinical course with no reported recurrences or metastatic disease, although its long-term malignant potential is uncertain.

**Case presentation:**

We present a 57-year-old man with an incidentally growing segment VII lesion that displayed features concerning for a neoplasm. He underwent a posterior sectionectomy and final histopathology confirmed a 27 mm HSVN. As the long-term malignant potential of HSVN is uncertain, he will be followed up with serial MRI scans over the next 5 years.

**Discussion:**

HSVN is a recently described entity, it is uncertain what the long-term malignant potential of HSVN can be. Reported cases have not shown any evidence of disease progression.

**Conclusion:**

There are no clear guidelines established on the duration of the follow-up of HSVN. More research needs to be done to determine further the natural history of these tumours and possible radiological criteria.

## Highlights

• Hepatic small vessel neoplasm is a recently described neoplasm of the liver.
• There is uncertain long-term potential.
• There are few cases reported to guide in surveillance and follow up.
• There are no reported recurrences or metastatic disease.

## 1 Introduction

Hepatic vascular tumours constitute a continuum from benign haemangiomas to highly malignant angiosarcomas. They can be diagnostically challenging for clinicians due to their rarity.

Cavernous haemangioma is the most common mesenchymal tumour of the liver and has a benign course. In comparison, angiosarcoma is aggressive, with a high recurrence rate and dismal survival, with the median survival is only six months after surgery [1,2]. Hepatic small vessel neoplasm (HSVN) is a recently identified low-grade vascular neoplasm, first described by Gill et al. in 2016 [3]. HSVN appears to be an incidental finding in adult patients. This neoplasm shows features of both AS and cavernous haemangioma; despite having an infiltrative growth pattern, there is minimal cytologic atypia and mitotic activity. These lesions also share *GNAQ* and *GNA14* mutations, which are also seen in several other vascular lesions [4].

Despite limited follow-up data, HSVN appears to demonstrate a benign clinical course, although its long-term malignant potential is unknown.

This case report has been reported in line with the SCARE criteria [5].

## 2 Case presentation

A 57-year-old man, while being investigated for biliary colic, was incidentally noted to have a complex cystic/solid liver lesion in segment VII of his liver. His medical and surgical history included atrial fibrillation, hypertension, and tonsillectomy as a child. He had no known allergies. He worked as a truck driver, was a non-smoker, and consumed alcohol on a social basis.

**Fig. 1:**
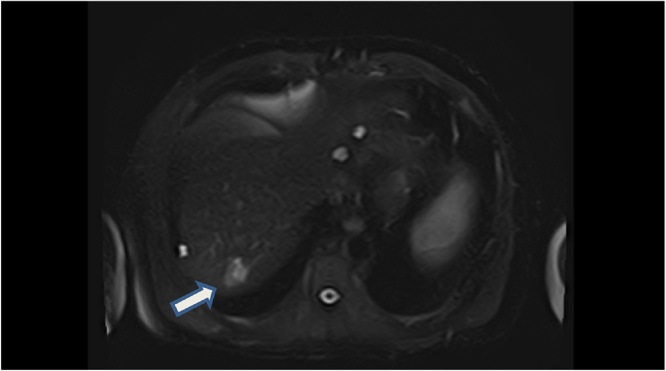
Axial T2 Fat-suppressed MRI image of liver. White arrow showing posterior segment VII lesion.

**Fig. 2:**
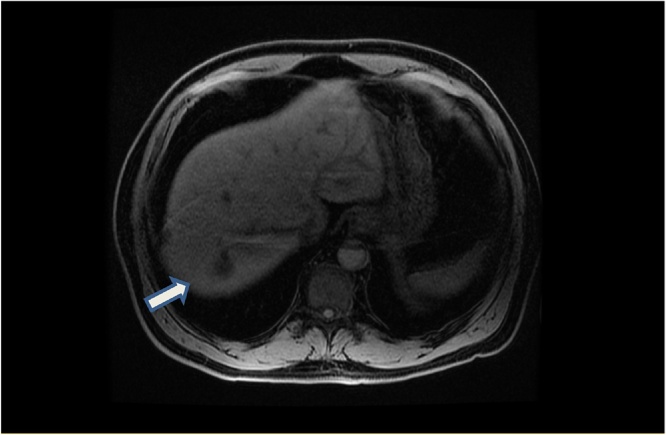
Axial LAVA Sequence (Three-dimensional spoiled gradient echo pulse sequence) of liver. White arrow showing posterior segment VII lesion.

Serial MRIs with gadoxetate disodium (Primovist) demonstrated mixed T2 signal intensity within the segment VII lesion, with arterial and portal venous phase enhancement, and no associated restricted diffusion (Fig. 1, Fig. 2). The solid component was isointense to the liver on the transitional phase with no retention of Primovist. The size of the lesion increased radiologically from 19 mm to 25 mm in 8 months. A separate 5 mm lesion in segment V was also noted, which had imaging characteristics of a possible haemangioma.

The patient proceeded to a laparoscopic posterior sectionectomy (segments VI and VII) with concomitant cholecystectomy. Intraoperatively, the lesion and simple liver cysts were identified on laparoscopic ultrasound. Surgery was uncomplicated, and he was discharged on day eight post-operatively.

**Fig. 3:**
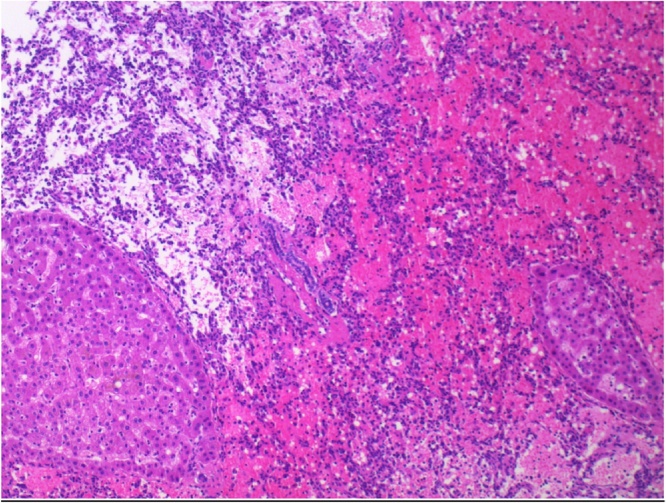
An intermediate-magnification view of the neoplasm, showing anastomosing vessels between residual hepatocyte islands (bottom left and bottom right). Despite infiltrative growth, there is no cytologic atypia and only rare mitotic activity.

Pathologic assessment of the resection specimen revealed a 27 mm poorly circumscribed unencapsulated mass, which appeared haemorrhagic macroscopically. Microscopically, the tumour was an infiltrative vasoformative lesion, with a degree of infiltration exceeding that of capillary or cavernous haemangioma. The tumour extended to the resection margin. There was no dissection between individual hepatocytes or cytological atypia (Fig. 3). On immunohistochemistry, the lesional cells showed a Ki-67 index of approximately 5%, p53 was wild-type (i.e. negative), and c-myc was negative. HSVN was suspected, and the diagnosis was subsequently confirmed after review by an expert gastrointestinal pathologist.

Initial follow up MRI at six months did not reveal any evidence of local recurrence. However, the previously noted segment V lesion had increased in size to 9 mm, raising the possibility of multifocal disease. Further surveillance imaging at 14 months revealed a stable appearance of the lesion. There was no local recurrence. Given the marginal growth and slow progression of the segment V lesion, and the uncertainty of the malignant potential of HSVN, the patient discussed at our multidisciplinary meeting with the decision to continue three-monthly MRI surveillance over the next five years.

## 3 Discussion

HSVN is a vascular neoplasm of the liver which can appear histologically similar to hepatic AS.

HSVN was defined as a distinct entity by Gill et al. in a case series of 17 vasoformative liver tumours collected over five years [3]. In this report, the average tumour size was 2.1 cm (range 0.2–5.5 cm), and gross examination showed poorly circumscribed pale tan to hemorrhagic lesions. On histologic assessment, the tumours were poorly circumscribed and featured infiltration of hepatic parenchyma by anastomosing capillaries, which were lined by bland endothelial cells. Immunohistochemical analysis showed uniform strong positivity for vascular markers (CD34, CD31 and FLI-1). Proliferative fraction, as measured by Ki-67, was low (3.7%). Molecular analysis was performed in three cases. Two tumours demonstrated an activating hotspot GNAQ mutation, with one of these tumours also showing an activating mutation in PIK3CA. HSVN and hepatic anastomosing hemangioma are vascular tumours have been shown to have the same activating mutations; however, the distinct infiltrative growth pattern seen in HSVN raises concerns for angiosarcoma [2].

Since the initial description by Gill et al., it appears four additional cases of HSVN have been reported, including our current case, giving a total of 22 cases [[6], [7], [8]].

HSVN are usually incidentally found in adult patients with a male preponderance and can range in size from 0.2 to 15.9 cm. The largest reported example occupied the whole left liver extending into the anterior section of the right liver [8].

Being a recently described entity, it is uncertain what the long-term malignant potential of HSVN can be. In the original description by Gill et al. [3] follow up data was available in 12 patients and, despite residual disease in some patients, there appeared to be no evidence of disease progression. However, follow up was limited (up to 5 months) in this subset of patients. Our patient had ongoing follow up at 14 months with no evidence of recurrence. Because of the uncertain malignant potential of HSVN and concern regarding the separate segment V lesion, the patient will have ongoing surveillance imaging over the next five years.

## 4 Conclusion

HSVN provides a follow-up conundrum for clinicians as it is a newly described entity with uncertain malignant potential. Clear guidelines need to be established on the duration of the follow-up of HSVN. More research needs to be done to determine further the natural history of these tumours and possible radiological criteria.

## Conflicts of interest

None to declare.

## Sources of funding

None to declare.

## Ethical approval

Ethical approval was not required for an anonymised case report.

## Consent

Written informed consent was obtained from the patient for publication of this case report and accompanying images. A copy of the written consent is available for review by the Editor-in-Chief of this journal on request.

## Author contribution

Patricia Mulholland - study concept or design, data collection, data analysis or interpretation, writing the paper.

Ian Y Goh - study concept or design, data collection, data analysis or interpretation, writing the paper.

Anna Sokolova - study or design, data collection, data analysis or interpretation, edition and final approval.

Cheng Liu - study concept or design, data collection, data analysis or interpretation, edition and final approval.

Mehan Siriwardhane - study concept or design, data collection, data analysis or interpretation, edition and final approval.

## Registration of research studies

Not applicable.

## Guarantor

Ian Y. Goh.

## Provenance and peer review

Not commissioned, externally peer-reviewed.
